# Tobacco retailer density surrounding schools and youth smoking behaviour: a multi-level analysis

**DOI:** 10.1186/1617-9625-9-9

**Published:** 2011-07-27

**Authors:** Wing C Chan, Scott T Leatherdale

**Affiliations:** 1 Department of Health Studies and Gerontology, University of Waterloo, 200 University Avenue West, Waterloo, Ontario, N2L 3G1, Canada

## Abstract

**Background:**

Youth smoking prevention should be a public health priority. It is not only vital to prevent youth from smoking but also to prevent non-smoking youth from becoming susceptible to smoking. Past research has examined factors associated with youth's susceptibility to become a future smoker, but research has yet to examine tobacco retailer density and susceptibility to smoking among never smokers. The objectives of this study are to examine how tobacco retailer density surrounding schools and social smoking influences are associated with smoking susceptibility among youth of never smokers, and occasional and daily smoking among youth of current smokers.

**Methods:**

Data were collected in 2005-2006 from grade 9 to 12 students attending 76 secondary schools in Ontario, Canada, as part of the SHAPES-On study. A series of multi-level logistic regression analyses were performed to understand how student- and school-level factors are associated with three smoking behaviour outcomes: smoking susceptibility among never smokers, occasional smoking, and daily smoking.

**Results:**

The number of tobacco retailers surrounding a school was found to be associated with the likelihood of a never smoker being susceptible to future smoking (OR 1.03, 95CI% 1.01, 1.05). We also identified that being surrounded by smoking social influences, specifically family and close friends, can substantially increase the likelihood that never smokers are at risk for future smoking or that youth are already occasional or daily smokers.

**Conclusions:**

We identified that the number of tobacco retailers surrounding a school was associated with an increased odds of being susceptible to future smoking among male never smokers. Smoking social models surrounding youth also appears to have an important impact on their smoking behaviour regardless of their smoking status. It is important for youth smoking prevention programs to begin early, interrupt youths' susceptibility to future smoking, and focus on subgroups that are at higher risk of smoking. The government should consider the impact of tobacco retailer density on youth smoking behaviour, and be cautious when granting licenses for establishments to sell tobacco products.

## Background

The prevalence of current smoking among youth aged 15-17 in Canada was 10% in 2008 [1]. This is cause for concern as youth who smoke are more likely to smoke as adults [2], and more likely to engage in other health risk behaviours [3-6]. Youth smoking prevention should be a public health priority.

Youth smokers can generally be categorized as being non-smokers, occasional smokers, or daily smokers [7,8]. Research has previously identified factors associated with occasional and daily smoking among youth, such as being male, perceptions of cigarette accessibility, use of drugs and alcohol, and influence from family and friends smoking [3,8,9]. Among non-smokers, research has examined factors associated with their susceptibility to become a future smoker [10-14]. Smoking susceptibility is defined as the absence of a determined decision not to smoke [13]. Although studies have shown that increased exposure to family and friends who smoke is associated with increased smoking susceptibility for youth [15-18], there is a paucity of research examining the characteristics associated with being susceptible. Considering that tobacco retailer density is a determinant of availability of tobacco products, and availability is a determinant of smoking behaviour [19], additional research examining how tobacco retailer density is associated with smoking onset and progression is needed. Research has previously identified that higher tobacco retailer density surrounding schools is associated with higher prevalence of occasional and daily smoking among students [20-22]. Research has yet to examine tobacco retailer density and susceptibility to smoking among never smokers. The current study builds on existing research by examining how tobacco retailer density surrounding schools is associated with smoking susceptibility among never smokers, and by further exploring if tobacco retailer density is associated with occasional or daily smoking. We also examined social smoking influences on youths' smoking susceptibility and smoking behaviour.

## Methods

### Design

This cross-sectional study used self-reported data collected in 2005-2006 from grade 9 to 12 students attending 76 secondary schools in Ontario, Canada, as part of the SHAPES-On study. Student-level data were collected from consenting students using the Tobacco Module (TM). Additional details about the SHAPES-On study and the TM are available online http://www.shapes.uwaterloo.ca/projects/SHAPES-ON. School-level data on tobacco retailers were provided by the Enhanced Points of Interest data resource from the Desktop Mapping Technologies Inc. (DMTI-EPOI) http://www.dmtispatial.com. School-level data on the socioeconomic status of the community in which the schools are located were derived from the 2006 Canadian Census http://www12.statcan.ca/census-recensement/index-eng.cfm.

### Data Collection

All surveys were completed in class time and participants were not provided compensation. Active information with passive consent was used to reduce demands on schools and increase student participation rates. The researcher informed the parents of the students via mail, and asked parents to call a toll-free number (accessible 24 hours a day) if they wanted to exclude their child from the study. The University of Waterloo Office of Research Ethics and appropriate School Board ethics committees approved all procedures, including passive consent.

### Participants

Of the 36,175 students eligible to complete the TM in participating schools, 74.4% (n = 26,924) completed the survey (missing respondents resulted from absenteeism on the day of the survey and parent/student refusal), and 71.6% (n = 25,893) provided complete data for this study.

### Measures

Smoking stage categories were consistent with existing research [8,13,23]. Consistent with a study by Pierce et al. (1996) [13], smoking susceptibility among never smokers (never smoked a cigarette, not even a puff) was determined from answers to three questions: (a) "Do you think in the future you might try smoking cigarettes?", (b) "If one of your best friends were to offer you a cigarette, would you smoke it?", and (c) "At any time during the next year do you think you will smoke a cigarette?" Only never smokers who answered 'definitely not' to all three questions on a 4-point Likert scale were considered non-susceptible [13]. Among students who reported smoking more than 100 cigarettes in their life, those who reported smoking tobacco everyday or almost everyday in the 30 days preceding the survey were considered daily smokers, whereas those who reported smoking some days or only 1 or 2 days in the 30 days preceding the survey were considered occasional smokers. Consistent with Health Canada guidelines [24], students who have smoked fewer than 100 cigarettes in their lifetime but have smoked in the 30 days preceding the survey were excluded from the analyses due to being experimental rather than established smokers (n = 752). Respondents also reported their grade, gender, if they had an older sibling who smokes, if they have a parent who smokes, and how many of their five closest friends smoke.

The *number of tobacco retailers *surrounding a school was determined using the 2005-2006 DMTI-EPOI data file. Consistent with previous research [25], linking the DMTI-EPOI data to the SHAPES-On student level data involved three steps: (1) geocoding the address for each SHAPES-On school; (2) creating 1-km circular buffers (i.e., bounded areas surrounding each school in which the number of tobacco retailers were quantified); and (3) linking the school-level tobacco retailer density for each school to the student-level data from each school. Arcview 3.3 [26] software was used to geocode the school addresses and to create the 1-km buffers. In order to control for the potential relationship between retailer density and the socioeconomic status of the community in which each schools is located, we used data from the 2006 census to identify the percentage of families in the community receiving government transfer payments (e.g., social assistance from provincial and municipal programs) as a measure of *neighbourhood disadvantage*.

### Analyses

Descriptive statistics of the student-level data were examined by gender. Using the school-level data, we calculated the mean and range for the number of tobacco retailers located within a 1-km radius participating schools. Since students (level-1) are nested within schools (level-2), a series of multi-level logistic regression analyses were performed to examine how student- and school-level factors were associated with our three smoking behaviour outcomes: smoking susceptibility among never smokers (Model 1); occasional smoking (Model 2); and daily smoking (Model 3). Consistent with other multi-level studies [23,27], a four step modelling procedure was used for each outcome. In Step 1, a random model effect was used to examine if differences in the outcome were random or fixed across schools. In Step 2, we examined if the number of tobacco retailers at the school-level was associated with the outcome as fixed effects, controlling for neighbourhood disadvantage. In Step 3, we examined how the student-level characteristics and the number of tobacco retailers at the school-level were associated with the outcome, controlling for neighbourhood disadvantage. In Step 4, contextual interactions between the student characteristics and the number of tobacco retailers at the school-level were examined. Statistical analyses were conducted on ML*wiN *Version 2.02 [28].

## Results

### Descriptive statistics

Descriptive results are presented in Table 1. Although the prevalence of daily smoking was similar for males and females, slightly more females than males were considered occasional smokers (χ^2 ^= 7.29, *df *= 2, p = 0.026). Of the 15,361 students classified as never smokers (never smoked, not even a puff), 4,539 (29.5%) were considered susceptible to future smoking. Female never smokers were more likely to be susceptible than males (28.5% vs. 30.7% respectively, χ^2 ^= 8.48, *df *= 1, p = 0.004). The mean number of tobacco retailers within a 1-km buffer of the schools was 2.68 (range, 0 to 16). As a measure of neighbourhood disadvantage, the mean percent of families receiving government transfer payments in the communities in which the schools were located was 9.6% (range, 3.6% to 16.8%).

**Table 1:** Descriptive statistics for youth in grades 9 to 12 by gender

		Total (n = 25,893)		Male (n = 13,206)		Female (n = 12,687)		Chi-Square
***Student-Level Characteristics***		%	(n)^a^	%	(n)^a^	%	(n)^a^	
Smoking Status	Daily smoker	9.5	(2,377)	9.5	(1,214)	9.4	(1,163)	χ^2 ^= 7.29, *df *= 2, p = 0.026
	Occasional smoker	8.9	(2,242)	8.4	(1,077)	9.4	(1,165)	
	Non-smoker	81.6	(20,522)	82.1	(10,470)	81.2	(10,052)	
Susceptibility to smoking	Susceptible	29.5	(4,539)	28.5	(2,284)	30.7	(2,255)	χ^2 ^= 8.48, *df *= 1, p = 0.004
(*never-smokers only*)	Not Susceptible	70.5	(10,822)	71.5	(5,724)	69.3	(5,098)	
Has an older sibling who smokes	Yes	29.0	(4,998)	28.1	(2,485)	30.0	(2,513)	χ^2 ^= 7.98, *df *= 1, p = 0.005
	No	71.0	(12,244)	71.9	(6,390)	70.0	(5,854)	
Has a parent who smokes	Yes	38.8	(10,049)	38.2	(5,047)	39.4	(5,002)	χ^2 ^= 3.98, *df *= 1, p = 0.046
	No	61.2	(15,844)	61.8	(8,159)	60.6	(7,685)	
Number of close friends who smoke	None	61.9	(14,599)	58.7	(7,635)	55.3	(6,964)	χ^2 ^= 53.80, *df *= 5, p < 0.001
	1	8.1	(1,922)	14.7	(1,916)	15.9	(2,006)	
	2	10.9	(2,581)	9.4	(1,224)	10.8	(1,357)	
	3	7.0	(1,659)	6.1	(799)	6.8	(860)	
	4	4.5	(1,058)	.7	(479)	4.6	(579)	
	5	7.6	(1,787)	7.4	(957)	6.6	(830)	
Grade	9	27.7	(7,168)	27.7	(3,664)	27.6	(3,504)	χ^2 ^= 11.24, *df *= 3, p = 0.011
	10	26.9	(6,974)	27.1	(3,575)	26.8	(3,399)	
	11	23.5	(6,085)	22.7	(3,001)	24.3	(3,084)	
	12	21.9	(5,666)	22.5	(2,966)	21.3	(2,700)	

^a ^Numbers may not add to total because of missing values or excluded cases

### Factors associated with smoking susceptibility

**Figure 1:**
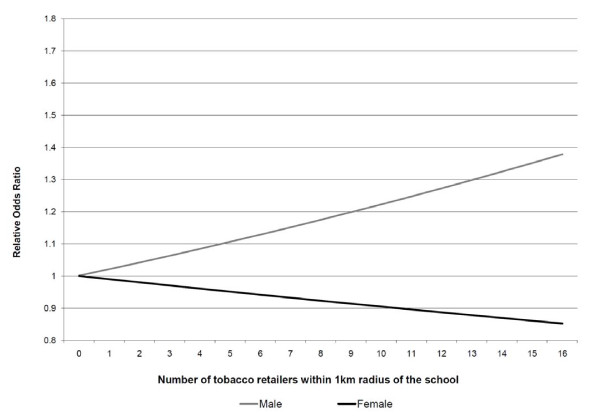
**Odds of a non-smoking student susceptible to smoking by number of tobacco retailers and sex**. Using the model estimates, the odds of a non-smoking student being susceptible to smoking can be estimated as a function of both the number of tobacco retailers within a 1-km radius of the school and the sex of the student. In Figure 1, the model-based odds ratios of a non-smoking student being susceptible to smoking relative to a hypothetical female student who attends a hypothetical school with no tobacco retailers within a 1-km radius are presented.

Results of the multi-level logistic regression analyses are presented in Table 2. Among never smokers, significant between-school random variation in the odds of being susceptible to smoking was identified [σ^2^_μ0 _= 0.06(0.01), p < 0.001]; school-level differences accounted for 1.8% of the variability in smoking susceptibility among never smokers. The odds of a never smoker being susceptible to future smoking increased with each additional tobacco retailer located within a 1-km radius of his/her school (OR 1.03, 95%CI 1.01 to 1.05); although a significant contextual interaction was identified with gender (β-0.04, p < 0.001). As shown in Figure 1, as the number of tobacco retailers surrounding a school increased, the relative odds of a male never smoker being susceptible to smoking increased and a female never smoker being susceptible to smoking decreased. While this increase in relative odds of being susceptible to smoking seen among male never smokers was statistically significant, the decrease seen among female never smokers was not. The neighbourhood disadvantage value for a school was not significantly associated with the likelihood of a never smoker being susceptible to smoking.

**Table 2:** Multi-level logistic regression analyses examining factors associated with youth smoking behaviour (grades 9 to 12)

		Adjusted Odds Ratio ^§^ (95% CI)		
		Model 1 Susceptible **vs**. Non-susceptible	Model 2 Occasional smoker **vs**. Non-smoker	Model 3 Daily smoker **vs**. Non-smoker
**Student-Level Characteristics**				
Sex	Female	1.00	1.00	1.00
	Male	0.93 (0.85, 1.01)	0.92 (0.82, 1.03)	1.12 (0.98, 1.28)
Has an older sibling who smokes	No	1.00	1.00	1.00
	Yes	1.17 (1.04, 1.31)**	1.81 (1.61, 2.03)***	2.51 (2.19, 2.87)***
Has a parent who smokes	No	1.00	1.00	1.00
	Yes	1.07 (0.97, 1.18)	1.23 (1.10, 1.39)**	1.89 (1.65, 2.17)***
Number of close friends who smoke	None	1.00	1.00	1.00
	1 to 2 friends	1.83 (1.65, 2.04)***	4.93 (4.29, 5.66)***	5.70 (4.47, 7.26)***
	3 or more friends	2.58 (2.17, 3.05)***	11.52 (9.89, 13.42)***	82.60 (66.19, 103.08)***
Grade	9	1.00	1.00	1.00
	10	0.77 (0.69, 0.86)***	1.19 (1.01, 1.41)*	1.70 (1.39, 2.08)***
	11	0.57 (0.50, 0.64)***	1.56 (1.32, 1.83)***	1.79 (1.46, 2.19)***
	12	0.43 (0.37, 0.49)***	2.01 (1.71, 2.36)***	3.25 (2.68, 3.96)***
**School-Level Characteristics**				
Number of tobacco retailers	Each retailer	1.03 (1.01, 1.05)*	0.99 (0.97, 1.01)	1.00 (0.98, 1.02)
Neighbourhood disadvantage	Each % increase	0.98 (0.94, 1.02)	0.98 (0.95, 1.00)	0.99 (0.97, 1.01)

Note: § Odds ratios adjusted for all other variables in the table.

Model 1: 1 = Susceptible never smoker (n = 4,539), 0 = Non-susceptible never smoker (n = 10,822)

Model 2: 1 = Occasional smoker (n = 2,242), 0 = Non-smoker (n = 20,522)

Model 3: 1 = Daily smoker (n = 2,377), 0 = Non-smokers (n = 20,522)

*p < .05 **p < .01 ***p < .001

A never smoker with an older sibling, 1 to 2 close friends, or 3 or more close friends who smoke(s) was more likely to be susceptible than a never smoker with no older siblings or close friends who smoke (OR 1.17, 95%CI 1.04 to 1.31; OR 1.83, 95%CI 1.65 to 2.04; OR 2.58, 95%CI 2.17 to 3.05 respectively). Compared to never smokers in grade 9, never smokers in grade 10, grade 11, and grade 12 were less likely to be susceptible to future smoking (OR 0.77, 95%CI 0.69 to 0.86; OR 0.57, 95%CI 0.50 to 0.64; OR 0.43, 95%CI 0.37 to 0.49; respectively).

### Factors associated with occasional smoking

Significant between-school random variation in the odds of being an occasional smoker was identified [σ^2^_μ0 _= 0.07(0.02), p < 0.001]; school-level differences accounted for 2.1% of the variability in occasional smoking. The number of tobacco retailers surrounding a school and the neighbourhood disadvantage value for a school were not significantly associated with being an occasional smoker.

A student with an older sibling, a parent, 1 to 2 close friends, or 3 or more close friends who smoke(s) was more likely to be an occasional smoker than a student with no older siblings, parents, or close friends who smoke (OR 1.81, 95%CI 1.61 to 2.03; OR 1.23, 95%CI 1.10 to 1.39; OR 4.93, 95%CI 4.29 to 5.66; OR 11.52, 95%CI 9.89 to 13.42 respectively). Students in grade 10, grade 11, and grade 12 were more likely to be occasional smokers than students in grade 9 (OR 1.19, 95%CI 1.01 to 1.41; OR 1.56, 95%CI 1.32 to 1.83; OR 2.01, 95%CI 1.71 to 2.36; respectively).

### Factors associated with daily smoking

Significant between-school random variation in the odds of being a daily smoker was identified [σ^2^_μ0 _= 0.25(0.04), p < 0.001]; school-level differences accounted for 7.1% of the variability in daily smoking. The number of tobacco retailers surrounding a school and the neighbourhood disadvantage value for a school were not significantly associated with being a daily smoker.

A student with an older sibling, a parent, 1 to 2 close friends, or 3 or more close friends who smoke(s) was more likely to be a daily smoker than a student with no older siblings, parents, or close friends who smoke (OR 2.51, 95%CI 2.19 to 2.87; OR 1.89, 95%CI 1.65 to 2.17; OR 5.70, 95%CI 4.47 to 7.26; OR 82.60, 95%CI 66.19 to 103.08 respectively). Similarly, students in grade 10, grade 11, and grade 12 were more likely to be daily smokers relative to students in grade 9 (OR 1.70, 95%CI 1.39 to 2.08; OR 1.79, 95%CI 1.46 to 2.19; OR 3.25, 95%CI 2.68 to 3.96; respectively).

## Discussion

Progress in reducing the prevalence of youth smoking will require efforts from many different stakeholders in many different contexts. While school-based interventions alone will not be sufficient to solve the problem, our results suggest that the school environment continues to represent one of the key contexts for intervening, as we identified significant between-school variability in all three of the smoking outcomes examined. Developing a better understanding of the modifiable school- and student-level factors associated with smoking among youth is critical for informing future programs and policies.

For example, to the best of our knowledge, this is the first study to identify that smoking susceptibility among students was positively associated with tobacco retailer density surrounding a school. Although the effect size of this association identified may appear modest relative to the variability which would be accounted for by characteristics at the student-level, it does represent a substantial amount of variation which could be amenable to intervention. Especially when one considers that even a modest school-level effect could impact a substantial number of youth when distributed across the broader student population. This new finding is important as research has previously suggested that perceived ease of access to tobacco is associated with smoking experimentation [24,29] and susceptibility [11]. If tobacco retailer density does influence perceived availability of tobacco products among subpopulations of youth, our results suggest that prevention activities should be targeted to the non-smoking students who are at the greatest risk at the school-level (i.e., male students attending a school surrounded by a high number of tobacco retailers). Such a targeted approach to prevention programming would require evaluation. Our result also suggests that it may be wise for decision makers to develop and evaluate policies that prevent tobacco retailers from being located within close proximity to schools as a starting point of a community-level intervention. A zoning law may be useful to keep tobacco products away from schools, and by extension youth, and thus reduce actual and perceived ease of access. In fact, one-third of youth smokers would smoke fewer cigarettes if they had to travel further to purchase them [30]. Such an approach is currently being used in some jurisdictions as a strategy for reducing consumption of alcohol [20,21]. Its effectiveness in tobacco use prevention among youth warrants implementation and evaluation in more jurisdictions, but there should be further consideration from the government when deciding the density, proximity, and types of establishments that are licensed to sell tobacco products.

Although research has previously identified that tobacco retailer density near a school is associated with an increased prevalence of smoking youth at a school [20,21], we did not identify an association between the odds of an individual student being an occasional or daily smoker and tobacco retailer density. This finding is consistent with the study by Leatherdale and Strath (2007) [22]. However, since we did identify that there was significant variability in occasional and daily smoking across schools, additional research is required to determine the modifiable schools characteristics (e.g., programs or policies), which may explain this variability. One way to accomplish this goal is to ensure that future school-based surveillance activities collect school-level program and policy data as well as student-level data.

Consistent with theoretical [31] and empirical research [9,32-34] highlighting the role of social influences on smoking onset, we identified that youth with a parent or sibling who smokes was more likely to be in an advanced stage of smoking behaviour in each of our analytical models tested. This suggests that smoking prevention programs should continue to target activities toward modifying the influence of family and friends who smoke on youth smoking.

While it may seem counter-intuitive how we identified that older non-smoking youth are less likely to be susceptible, it is likely due to the artefact of older students having already tried or started smoking; hence they are no longer considered susceptible. We also identified that although non-smoking females were more likely to be susceptible than non-smoking males, their likelihood of being susceptible decreased as tobacco retailer density increased (as shown in Figure 1). Although it can not be determined with these data, it may be due to female smokers being less likely to purchase their own cigarettes [35], and more likely to seek alternative sources such as male friends, family members, or strangers. While this decrease in likelihood of susceptibility among non-smoking females is not statistically significant, this unique association deserves further investigation.

This study is subject to limitations common to survey research. The cross-sectional nature of the design does not allow for causal inferences from the associations identified in this study. There is no information on the reliability and validity of the DMTI-EPOI data. Different studies have examined either tobacco retailer density or distance to the nearest tobacco outlet on youth smoking outcomes. The use of tobacco retailer density in this study is based on previous research conducted by Leatherdale and Strath (2007) [22]. We did examine the number of tobacco retailers within a 1-km buffer of the schools, which is within close walking distance for youth to access tobacco products in these outlets. The association between tobacco retailer density and smoking susceptibility among youth of non-smokers is significant but the amount of variability is modest. We believe there may be unmeasured confounders at the school-level that are associated with youth smoking behaviour which were not measured in this study (e.g., school-based prevention programs and policies). We controlled for neighbourhood disadvantage in the analysis by using the mean percent of families receiving government transfer payments in the communities in which the schools were located. Additional measure of socioeconomic status of the community may be warranted in future studies.

## Conclusions

The current study examined the association between tobacco retailer density and smoking susceptibility among never smokers, and occasional and daily smoking among current smokers. We identified that the number of tobacco retailers surrounding a school was associated with an increased odds of being susceptible to future smoking among male never smokers. Smoking social models surrounding youth also appears to have an important impact on their smoking behaviour regardless of their smoking status. It is important for youth smoking prevention programs to begin early, interrupt youths' susceptibility to future smoking, and also focus on subgroups that are at higher risk of smoking such as male youth and youth with family and friends who smoke. Moreover, the government should consider the impact of tobacco retailer density on youth smoking behaviour and health, and be cautious when granting licenses for establishments to sell tobacco products.

## Competing interests

The authors declare that they have no competing interests.

## Authors' contributions

STL conceived the idea for the study and performed the statistical analyses. WCC derived the school-level data on the socioeconomic status of the community and wrote the first draft. Both authors read and approved the final version of the manuscript.
